# Genome Sequence of *Gordonia rubripertincta* Phage DoobyDoo of the DV Cluster

**DOI:** 10.17912/micropub.biology.001471

**Published:** 2025-03-15

**Authors:** Ibrahim Abdulrehman, Nathaly Angeles, Alexis Bostock, Ciara Burns, Srey Lim, Adrienne Nalley, Madison Olander, Preston Penny, Bailey Phipps, Jack Price, Jacob Rose, Gabrial Smith, Mackenzie Spalding, Sadie Weisman-Rosenberger, Pamela Connerly, Danielle Watt, Elizabeth Rueschhoff

**Affiliations:** 1 School of Natural Sciences, Indiana University Southeast, New Albany, Indiana, United States; 2 School of Social Sciences, Indiana University Southeast, New Albany, Indiana, United States; 3 School of Arts and Letters, Indiana University Southeast, New Albany, Indiana, United States

## Abstract

Novel bacteriophage DoobyDoo was isolated and characterized utilizing host *Gordonia rubripertincta* NRRL B-16540. DoobyDoo has a siphoviral morphology and a 66,343 bp genome with a GC content of 58.3%. The genome contains 97 protein coding genes, including an esterase gene that is distributed broadly across actinobacteriophages.

**Figure 1. DoobyDoo plaques and particles:**
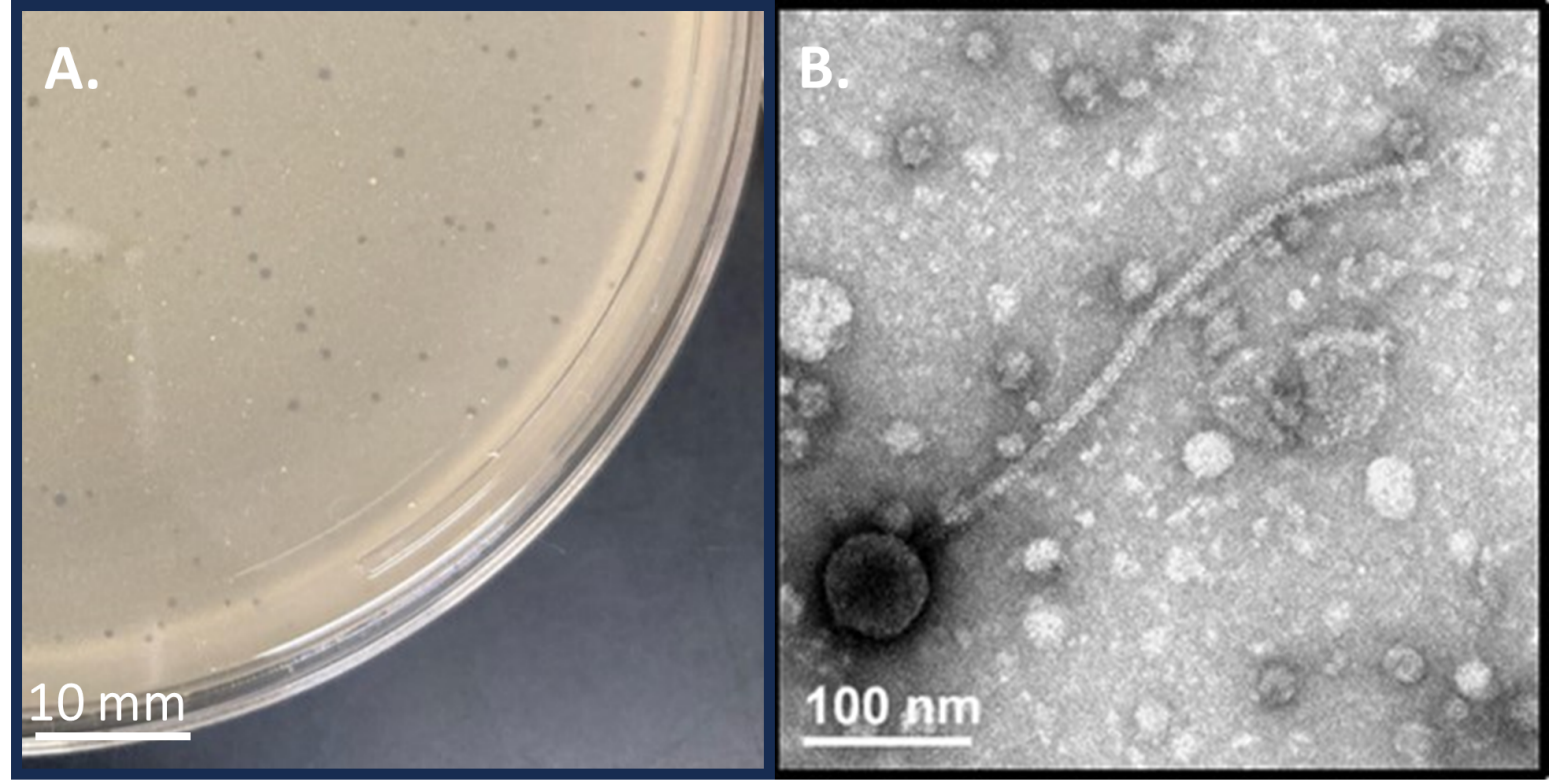
(A) Plaques of DoobyDoo are approximately 1 mm in diameter and clear, with irregular margins. (B) Negative stain (uranyl acetate, 2%) transmission electron microscopy reveals a siphovirus bacteriophage with a capsid diameter of 65.6 nm +/- 1.9 nm and tail length of 388 nm +/- 11.5 nm (n=5). Capsid and tail sizes were measured with ImageJ software (Schneider et al. 2012).

## Description

Bacteriophage genomes represent a vast reservoir of uncharacterized genes. Phages capable of infecting *Gordonia* are a diverse group; as of February 2025, 842 *Gordonia* bacteriophages have been sequenced and they are grouped into 39 different clusters and 8 single-members (singletons), based on gene content similarity (GCS) of at least 35% to phages in the Actinobacteriophage database, phagesDB (Pope et al. 2017; Russell and Hatfull 2017). Isolating and characterizing *Gordonia* *sp.* bacteriophages provides an opportunity to explore the vastness of bacteriophage genetic diversity and their evolution.

DoobyDoo was isolated from soil collected from New Albany, IN, USA (Global Positioning System [GPS] Coordinates 38.358060, -85.802700), by enriched isolation. The soil sample was suspended in peptone yeast calcium (PYCa) medium, then filtered (0.2 µm filter). The filtrate was inoculated with *Gordonia* *rubripertincta* NRRL B-16540 and incubated at 26˚C for approximately five days. The culture was then refiltered, plated with *G. rubripertincta* using a soft agar overlay, and incubated at 26˚C for 48 hours. DoobyDoo formed clear, round plaques with irregular edges approximately 1 mm in diameter ( Figure 1A ). DoobyDoo was purified through two additional rounds of plating (Zorawik et al. 2024). Negative stain transmission electron microscopy (2% uranyl acetate) revealed a siphovirus morphology, with capsid size of 65.6 nm +/- 1.9 nm in width and tail length of 388 nm +/- 11.5 nm (n=5) ( Figure 1B ).

DNA was isolated from a lysate using a QIAGEN DNeasy Blood and Tissue Kit (Jakociune and Moodley 2018). DNA was prepared for sequencing using NEB Ultra FSII kit, and sequencing was performed using the Illumina platform (v3 reagents). There were 362,709 single-end 150 base reads yielding a 773-fold average coverage. Newbler v2.9 and Consed v29 were used to assemble the raw reads and check them for completeness (Russell 2018). Genome ends were circularly permuted (Russell 2018). The genome is 66,343 bp in length with a GC content of 58.3%. Based on GCS, DoobyDoo was placed in actinobacteriophage cluster DV, which includes phages isolated using *G. rubripertincta* and one phage isolated using *G. terrae* NRRL-B16283 (Pope et al. 2017; Russell and Hatfull 2017).

The genome was auto-annotated using Glimmer v3.02b and Genemark v2.5p (Delcher et al. 2007; Besemer and Borodovsky 2005). Manual annotation was performed using DNA Master v5.23.6 (http:// cobamide2.bio.pitt.edu), BLASTp v2.15.0 utilizing the NCBI nonredundant and Actinobacteriophage databases (Altschul et al. 1990), Starterator v574 (http://phages.wustl.edu/starterator/), Phamerator Actino_Draft v574 (Cresawn et al. 2011), HHPred utilizing the PDB_mmCIF70_24_Dec, Pfam-A_v36, UniProt-SwissProt-viral70_3_Nov_2021, and NCBI_Conserved_Domains(CD)_v3.19 databases (Söding et al. 2005), Aragorn v1.2.41 (Laslett and Canback 2004), tRNAscanSE v2.0.012 (Lowe and Eddy 1997), and DeepTMHMM v1.0.24 (Krogh et al. 2001). A total of 97 protein coding genes were predicted, with no tRNA genes. All genes are transcribed in the same direction.

Consistent with cluster DV phages, DoobyDoo encodes a putative metalloprotease (gene 10) within its structure and assembly genes. Within this region of the genome, there is a highly variable region in which DV phages encode esterase, lipase, or hydrolase functions. Here, DoobyDoo encodes a putative esterase (gene 4), homologues for which are found in phages that infect three different host bacteria and which belong to three different clusters. The diversity of phages in which this gene is found is congruent with the diverse nature of *Gordonia* sp. bacteriophages described by Pope et al. (2017). In addition, consistent with other phages which infect *G. rubripertincta* , DoobyDoo encodes its putative lysis genes across two regions of the genome; lysin A (gene 35) and lysin B (gene 86) are encoded in the central and terminal regions of the genome, respectively. No immunity repressor, integrase, excise, or cro functions were identified in DoobyDoo or other DV phages, suggesting these phages are unlikely to establish lysogeny.

**Nucleotide sequence accession numbers**

DoobyDoo is available at GenBank with Accession No. PQ362665 and Sequence Read Archive (SRA) No. SRX26311143.
